# Book Review: Innovations and Challenges in Language Learning Motivation

**DOI:** 10.3389/fpsyg.2020.593824

**Published:** 2020-09-30

**Authors:** Youqi Zheng, Chunhong Yang

**Affiliations:** Department of Foreign Languages, College of Liberal Arts, Nanjing University of Information Science and Technology, Nanjing, China

**Keywords:** language learning motivation, innovations, challenges, research frontiers, psychology

Language learning motivation researches have prospered for more than 50 years and shown that motivation is a multi-faceted notion affecting learning outcomes of target language. Despite the rich literature in this area, its complexity and multiple meanings result in some conflict findings, unexplored issues and challenges. *Innovations and Challenges in Language Learning Motivation* written by Zoltán Dörnyei, a leading scholar in L2 acquisition, offers an overview of research history of motivation with a focus on the latest challenges with corresponding solutions and uncovers some novel topics under-researched as well. As a language teacher and researcher in SLA area, I find it a useful reference book for my teaching and research.

This book consists of two parts: challenges and innovations (in the first three chapters) and research frontiers (in the last three chapters) which are closely connected. The first three chapters focus on the fundamental challenges, covering the following topics: the conceptualization of motivation, motivation dynamics, and motivation applied. Drawing on the rich literature, the author presents some innovative and conductive solutions to these challenges. The last three chapters make further elaboration on three issues which remain relatively unexplored. But as the author argues, research on these issues will make contributions to better understanding of language learning motivation in SLA and human behavior in general psychology.

Chapter one addresses the challenges and innovations of conceptualization of motivation which sets the scene for the following chapters. The state-trait dilemma and situational dependence of motivation raises the first “question of what motivation really is, a state, a trait or a process” (Dörnyei, 2020, p. 5). In terms of state-trait dichotomy, “New Big Five” model (McAdams and Pals, 2006), with a three-tier framework of personality provides theoretical framework to understand the trait-state distinction. However, this model has limited explaining power concerning the process-oriented nature of motivation, thus raising the second question of how to conceptualize motivation in a process-oriented manner. The author outlines some theoretical attempts including process models of motivation, contingent path theory, time perspective and the study of velocity in goal pursuit to tackle the time scales of motivation. It is obvious that these innovations shed light on the conceptualization of motivation.

Another challenge presented in this chapter is whether it is possible to distinguish motivation from cognition and affect since these three concepts are intricately intertwined. The author argues that the phenomenological account may provide support to separate them, in that motivation, cognition, and affect are associated with different consequences. Besides, the findings of latest neurobiological researches offer findings and evidence that can distinguish motivation and affect.

Chapter two makes a further analysis on the challenge of accounting for dynamic nature of motivation from the perspective of the process within the framework of complex dynamic systems theory. The author first attempts to deal with the challenges of appropriate theoretical handling of contextual factors in relation to individual characteristics by integrating some studies. Innovations 1-3 which include systematic characterizations of context, the rise of social motivation and qualitative research provide the timely and promising solutions. It is noteworthy to see the rise of “social motivation” researches in mainstream psychology, bridging “the person vs. environment divide in motivation research.” (Urdan, 2001, p. 171). Then, the application of qualitative methodology is of great help in understanding people's motivations and behaviors in local and social contexts and can offer rich data resources and thick description. Therefore, an increasing number of qualitative studies have been conducted to investigate the lived experiences of language learners in different contexts, thus enabling researchers and teachers to understand motivations and behaviors as they interact with social and contextual processes. Moreover, the author highlights impact of contextual factors in language learning motivation in innovation 4 by reviewing Gardner's motivation theory. In terms of researches in SLA, a situated analytical approach (Ushioda, 2009) highlighting person-in-context relational view of motivation stresses the agency of learners in complex social realities, which blurs the distinction between the agent and its context as well.

As for timescales of motivation, the researchers might encounter the challenges of what scale to use and how to measure motivational changes which are essential to understand motivation as a process. Accordingly, the ensuing three innovations, namely the idiodynamic method, appropriate time window and timescale and proximal subgoals, are outlined with some case study analysis. Then the author addresses the challenge of the interference of multiple parallel goals and argues that the dynamic system theory may offer some principles to tackle this challenge. Besides, goal configurations and temporal structuring and goal prioritization can shed lights on this challenge.

In Chapter three, the author turns to “practical applicability” (Dörnyei, 2020, p. 49) of the motivation research and explores three challenging areas. The first challenge presented is the limitation of the motivation research in SLA, which mainly focuses on language learning in general and less deals with the more specific processes of language acquisition. In response to “non-L2-specificity challenge” (Dörnyei, 2020, p. 51), the author proposes taking a “small lens approach” which means a “sharpening” of the empirical research focus (Ushioda, 2016) by narrowing down the scope of the study and focusing on the specific motivational topics in order to explore the impact of the motivation on the specific learning tasks such as reading and academic writing. In addition, the task-based motivation approach is of use in researching specific learning tasks of SLA by using the principles proposed by the author in student engagement and directed motivational currents.

As for the challenge of enhancing motivation, the author presents four innovations with four insightful methods of increasing motivation and makes a detailed analysis of how these ways work. The first innovation addressed is to adopt motivational strategies which provide useful guidance to both teachers and researchers. The second one is to emphasize students' engagement to ensure that the students' positive disposition is realized in action for motivation. Capitalizing role models is another way to motivate learners. It is noteworthy to see that learners in China and India tend to have non-native-speaking role models instead of native speakers. The fourth effective strategy is to prevent demotivation and remotivate learners. Then the author discusses the motivational potentials of technology and points out possible issues such as the novelty element in using technology, superficial engagement, and additional distractions, student reluctance and teacher reluctance.

The ensuing challenge is about how to measure dynamic process motivation. As for this challenge, the author argues that the traditional quantitative methods are not suitable to measure the dynamic concept and its relation to the contextual and temporal scales. Therefore, qualitative, longitudinal and intervention studies and mixed methods research are required to account for the dynamic complexity of motivation.

Based on challenges and innovations addressed in part I, the author makes a detailed analysis of three unexplored research frontiers in part II, thus offering promising directions and laying foundation for further studies in this area. Chapter four focuses on unconscious motivation and explores the following issues: human agency and its unconscious limits, unconscious goal setting and goal pursuit, and dual-process theories and interaction of the conscious and the unconscious mind and researching unconscious motivation. Obviously, the traditional measures are not effective in testing unconscious motives. The author outlines two areas of research methodology that are of profound significance in generating and identifying unconscious motives. On the one hand, technique of priming can help researchers investigate the features of unconscious mental processes. On the other hand, three research instruments have been considered useful to identify and assess unconscious attitudes and motivation. But we should be aware of the respective use of conscious and unconscious measures which provide implications for relevant research designs. Researchers should bear in mind the incremental value and dissonance vs. congruence between conscious and unconscious motives. Although this chapter makes contribution to the unconscious motivation, the relationship between conscious and unconscious motives still remains open and needs further study.

Chapter five shifts focus to motivating mechanisms of vision connected to the “faculty of mental imagery” (Dörnyei, 2020, p. 102) of human beings. After an overview of the meanings of vision in different contexts, the author points out the physical perception, future aspiration and mental picture are interlinked since they are all connected to the faculty of mental imagery. A historical summary of relevant studies in psychology sets the scene and the review of sports and business management elucidates the motivational power the vision possesses. Then the author outlines several channels through which the vision can impact human behavior from ideal self-images to positive emotions. In respect to emotions, it is argued that positive emotions contribute to the motivational processes. Therefore, part of vision's motivational power could be mediated by the emotions. Regarding vision and L2 motivation, the author discusses the application of vision in SLA and outlines some issues, ranging from the undertheorized nature of the L2 learning experience to the dilemma about negative imagery, which provides some new topics for the language teachers and researchers.

The following chapter discusses long-term motivation and persistence. A detailed analysis offers a broad framework of motivational processes and factors that together can stimulate long-term motivation. Interestingly, the term energy is used to show motivated behavior's power. The author elaborates on directed motivational currents and psychological momentum, contributing much to regenerating energy in sustaining long-term motivation. Then the author argues that four issues which are of significance in understanding long-term motivation deserve our attention and offers a theoretical construct with several components by using a vivid motoring metaphor.

Just as the author states in the concluding remarks, research on these new issues will serve a better understanding of language learning motivation in SLA. The reasons are as follows: (1) if we learn to harness the power of unconscious motives and attitudes, they may become assets for language learning; (2) the faculty of vision/ mental imagery may become a useful learning strategy and can provide more benefits ranging from memory enhancement to teaching language skills; (3) the key constituents of long-term motivation and their relationship should be identified to help learners grasp strategies and inspire their long-term commitment to learning.

All in all, drawing on impressive amounts of robust research both in mainstream psychology and language learning areas and the author's own researching experiences, the author makes a comprehensive and insightful overview of motivation researches with a focus on a broad spectrum of challenges and innovations which are amply analyzed throughout the book by offering a review of relevant literature, detailed analysis, theoretical bases and empirical findings. Hopefully this book has opened many venues and provided theoretical frameworks and practical solutions for future studies. It is destined to stimulate much productive further research. This is a must for teachers, postgraduate students and researchers interested in language learning and teaching. Researchers may find this book useful in delving deeply into this area. Due to the essential role motivation plays in successful language learning, it is of also high pedagogical value for language teachers to understand learners' differences and adopt appropriate motivational strategies to maximize their effectiveness. Besides, the list of references in each chapter provides further reading for those who would like to learn more.

## Author Contributions

YZ has written the review. CY revised the drafts. All authors contributed to the article and approved the submitted version.

## Conflict of Interest

The authors declare that the research was conducted in the absence of any commercial or financial relationships that could be construed as a potential conflict of interest.
